# Bone Response to Conventional Titanium Implants and New Zirconia Implants Produced by Additive Manufacturing

**DOI:** 10.3390/ma14164405

**Published:** 2021-08-06

**Authors:** Jin-Cheol Kim, In-Sung Luke Yeo

**Affiliations:** 1 Department of Prosthodontics, Seoul National University School of Dentistry, Seoul 03080, Korea; lyon3@snu.ac.kr; 2 Department of Prosthodontics, School of Dentistry and Dental Research Institute, Seoul National University, 101 Daehak-ro, Jongno-gu, Seoul 03080, Korea

**Keywords:** three-dimensional printing, zirconium oxide, osseointegration, titanium, bone-implant interface

## Abstract

The aim of the present study was to evaluate the in vivo bone response to an additively manufactured zirconia surface compared to osseointegration into titanium (Ti) surfaces. Scanning electron microscopy, confocal laser scanning microscopy, and electron spectroscopy for chemical analysis were performed to assess the surface characteristics of implant specimens. For the in vivo evaluation, eight Ti implants and eight 3D-printed zirconia implants were used. The surface of four Ti implants was sandblasted, large-grit, and acid-etched (Ti-SLA group), while those of the other four Ti implants were left untreated (Ti-turned group). The zirconia implants had no further surface modification. Implants were placed into the tibiae of four rabbits; two received the Ti-SLA and zirconia implants and the other two received Ti-turned and zirconia implants. The experimental animals were sacrificed after four weeks of surgery, and the undecalcified microscopic slides were prepared. The bone–implant interface was analyzed by histomorphometry to evaluate the bone response. The degree of surface roughness showed that Ti-SLA was the highest, followed by zirconia and Ti-turned surfaces. The 3D-printed zirconia surface showed similar bone-to-implant contact to the Ti-turned surface, and Ti-SLA had the most bone-to-implant contact. The additively manufactured zirconia implant surface is biocompatible with respect to osseointegration compared to the commercially pure Ti surface.

## 1. Introduction

Since its initial use in the clinical application of dental implants, over 40 years ago, titanium has been considered as a gold standard material in terms of mechanical strength, chemical inertness, and biocompatibility, and it has shown a high success rate [1,2]. However, several problems that impede the long-term success of the titanium implants have been reported. The gray color of titanium reduces patient satisfaction in terms of aesthetics [3,4,5,6]. Furthermore, some studies have reported allergic reactions, sensitivities, and corrosion of Ti [7,8,9]. 

The properties of zirconia (zirconium dioxide, ZrO_2_) dental implants have been investigated over the last 20 years to explore whether a zirconia implant can overcome the disadvantages of the titanium implant [10,11]. The advantage of zirconia implants over titanium is that the metal aura is invisible even in the case of buccal bone loss or/and the thin soft-tissue biotype. Zirconia is biocompatible and highly resistant to wear and corrosion [6,12]. However, zirconia is sensitive to low-temperature degradation (aging), which is vulnerable to subcritical bending and crack growth [13]. Therefore, the fracture resistance of zirconia is lower than that of titanium [14]. Implant success is evaluated by the degree of osseointegration at the bone-implant interfaces, which is expressed as the bone-to-implant contact ratio [11]. Several studies have shown that compared to titanium implants, zirconia implants have similar bone-to-implant contact rates [15,16].

Recently, interest in customized zirconia implants has increased due to the rapid development of computer-aided design and computer-aided manufacturing (CAD/CAM) technology [15]. Additive manufacturing (AM), better known as three-dimensional (3D) printing, is an additive production technique. This technique enables the fabrication of complex objects, such as an individualized layer-by-layer additive method, from computer-aided design (CAD) data, without the long production time and high cost of instruments and molds required in conventional milling technology [17]. Most of the zirconia implants studied through the existing milling technology are one-piece systems, and there are reports of higher crestal bone loss and low survival rates for a one-piece zirconia implant compared to a two-piece zirconia implant [18]. The customized additive manufacturing method can use a more sophisticated and diverse approach to produce zirconia-implant–abutment complexes than the conventional milling method. However, there have not been any in vivo studies evaluating the osseointegration of 3D-printed zirconia implants to date.

The aim of this study was to evaluate the bone response of titanium (machined and treated surface) versus zirconia implants, which were printed using AM methods inserted in rabbit tibiae. Furthermore, the physicochemical properties of 3D-printed zirconia implants were also analyzed. The null hypothesis underlying this study was that 3D-printed zirconia implants showed no significant difference in osseointegration compared to titanium implants.

## 2. Materials and Methods

### 2.1. 3D-Printed Zirconia Implant

With the lithography-based ceramic manufacturing process, zirconia implants (Lithoz, Vienna, Austria) were designed and 3D-printed. The zirconia implant possesses a content of 3 mol % yttrium oxide (3Y-TZP; Yttria-Stabilized Tetragonal Zirconia Polycrystal: LithaCon 3Y 230, Lithoz, Vienna, Austria). The macroscopic design of the titanium implants (Dentium, Seoul, Korea) was transferred to the experimental zirconia implants, which had the same shapes and dimensions (a standardized diameter of 3.8 mm and a length of 10 mm).

### 2.2. Surface Characterization

The implants’ surface was photographed by field emission-scanning electron microscopy (FE-SEM; S-4700, Hitachi, Tokyo, Japan). The element composition of each implant was performed by electron spectroscopy for the chemical analysis (ESCA; Sigma Probe, Thermo Scientific, Waltham, MA, USA). Surface parameters for the surface topography of the implants were measured by confocal laser scanning microscopy (CLSM; LSM 800, Carl Zeiss AG, Oberkochen, Germany). Each implant was analyzed at three different selected areas (top, middle, bottom), which were averaged and assigned to the representative value for one sample (top, [19]). (1) For the Sa (arithmetical mean height) value, the absolute values express the difference in the height of each point compared to the arithmetical mean of the surface. (2) The Sdr (developed interfacial area ratio) value shows the ratio between the definition area’s additional developed surface area and a flat definition area.

### 2.3. In Vivo Surgery

A total of sixteen screw-shaped implants were used in this study. Eight zirconia implants (Lithoz, Vienna, Austria) were applied as they were, without any surface modification. Eight titanium implants (Dentium, Seoul, Korea) were prepared and made of commercially pure titanium with/without surface modification (sandblasted, large-grit, acid-etched, or SLA surface/turned surface). Four female New Zealand white rabbits (age: 3–4 months; weight: 2.5–3.0 kg) were included in this study. This investigation was approved by the institutional animal research ethics committee of Cronex (CRONEX-IACUC: 202004001) and were conducted following the Animal Research: Reporting In Vivo Experiments (ARRIVE) guidelines [20]. Four rabbits were anesthetized via 1 mL of intramuscular injections, with a dose of 15 mg/kg tiletamine hydrochloride and zolazepam hydrochloride (Zoletil, Virbac, Carros, France) and 5 mg/kg xylazine (Rompun, Bayer AG, Leverkusen, Germany). The hind legs of the rabbits were shaved and sanitized with betadine. Infiltration anesthesia was performed at the surgical sites. By full-thickness incisions from the skin to periosteum, the medial side of both tibiae was exposed. The implant sites were prepared by rotating drills and engines under copious irrigation using a sterile saline solution. The final drill size was 3.4 mm. The implants were installed with primary stability ≥20 Ncm using a torque wrench (Dentium, Seoul, Korea). Each rabbit received four implants, meaning that the right and left tibia received two implants each. The zirconia and Ti-turned implants were inserted according to a 2 × 2 Latin square in two experimental animals. In the rest of the animals, the zirconia and SLA Ti implants were placed in the same way. At the surgical wound, the flaps were sutured with 4-0 vicryl (Ethicon, Somerville, MA, USA) and 4-0 Blue Nylon (Ailee, Busan, Korea). Postoperatively, antibiotic prophylaxis was administered using enrofloxacin (Biotril, Komipharm International, Siheung, Korea) to all the rabbits. The animals were housed in separate cages.

### 2.4. Histologic Assessment

At 4 weeks after implant installation, four rabbits were anesthetized and sacrificed by an intravenous overdose of potassium chloride for histologic assessment. The implants surrounding tissues and bones were surgically harvested en bloc. Each section was fixed in 10% neutral buffered formalin (Sigma-Aldrich, St. Louis, MO, USA) for 2 weeks, which was followed by dehydration with a graded series of ethanol, and then they were embedded in methyl methacrylate (Technovit 7200, Heraeus Kulzer, Hanau, Germany). The embedded blocks were sliced using an EXAKT cutting unit (EXAKT Appratebau, Norderstedt, Germany), following the methods described in previous studies [21,22,23], after which they were prepared with approximately 50 μm thickness and stained with Masson’s trichrome for examination using a light microscope. The interfaces between the bones and implants were examined to measure the degree of bone-to-implant contact (BIC) and bone area (BA) at the best consecutive threads. The histologic evaluation was performed using a light microscope (BX51, Olympus, Tokyo, Japan) on ×100 magnification connected to a CCD camera (SPOT Insight 2Mp scientific CCD digital camera system, Diagnostic Instruments, Sterling Heights, MI, USA) and an adaptor (U-CMA3, Olympus). The SPOT software version 4.0 (Diagnostic Instruments, Sterling Heights, MI, USA) and image analysis program (Image J 1.60, NIH, Bethesda, MD, USA) were used to analyze the acquired images.

### 2.5. Statistical Analysis

Most of the outcome variables for data normalization were accepted using the Shapiro–Wilk test (*p* > 0.05). Descriptive statistics are described using the mean and standard deviation. To analyze the difference in the surface of implants, the Ti-turned, Ti-SLA, and zirconia implants were compared using the analysis of variance (ANOVA). To assess the difference in BIC and BA values between Ti-turned, Ti-SLA, and zirconia implant, the paired *t* test was applied. The statistical software R (version 3.6.1, R Foundation for Statistical Computing, Vienna, Austria) was used for the analysis. Statistical significance was set at *p* < 0.05. 

## 3. Results

### 3.1. Surface Physical Analysis

**Figure 1:**
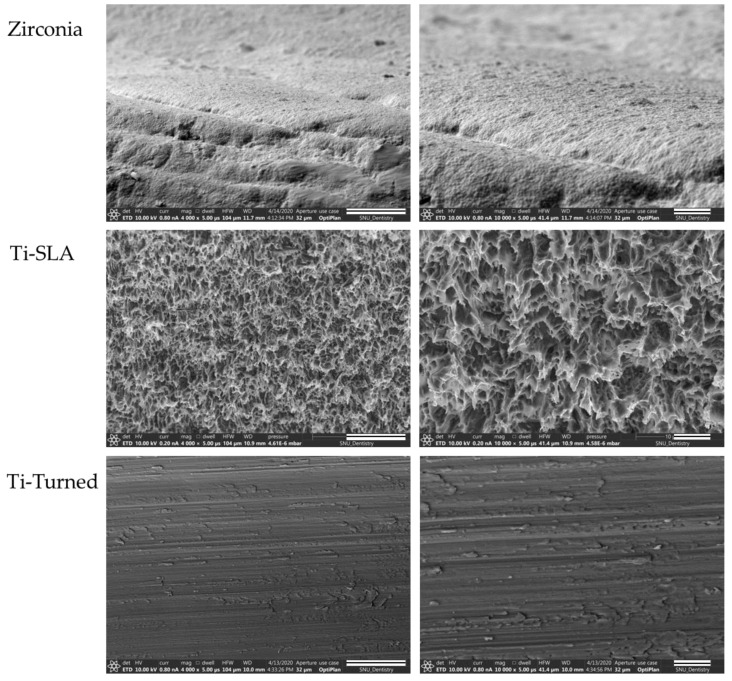
Field emission scanning electron microscopy (SEM) images of zirconia (top), Ti-SLA (middle), and Ti-turned implants (bottom) (magnification: ×4000 (scale bars, 20 μm) and ×10,000 (scale bars, 5 μm) from the left). Images demonstrated different surface morphologies among the Zirconia, the Ti-turned, and Ti-SLA implant surfaces.

Field emission scanning electron microscopy images demonstrated a different surface morphology among the Ti-turned, Ti-SLA, and zirconia implant surfaces (Figure 1).

Zirconia implants revealed several microcracks, porosities, and interconnected pores [24]. Ti-turned implants showed smooth, polished, and flat surface that run in one direction, while Ti-SLA implants had very rough, irregular, and honeycomb-like surfaces.

**Figure 2:**
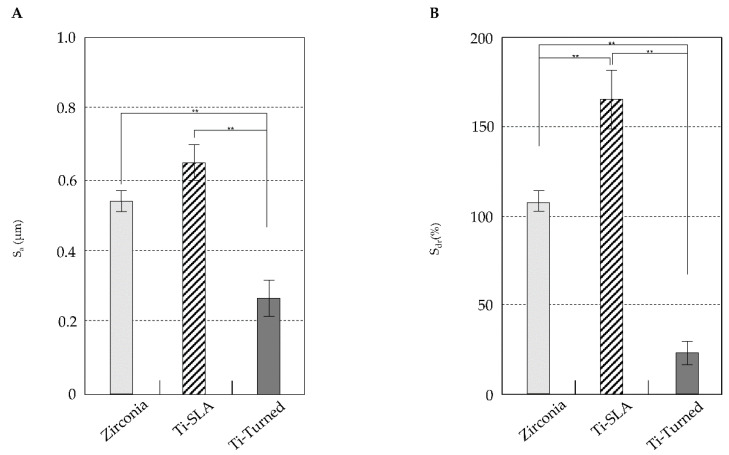
The data of roughness parameters (Sa, Sdr) of the groups. (**A**) The surfaces of the Ti-SLA and zirconia implants were significantly rougher than that of Ti-turned implants based on the Sa value (*p* < 0.01). Although the Sa value seemed to be higher for the Ti-SLA implants than zirconia implants, the difference was not statistically significant (*p* = 0.084). (**B**) The mean Sdr values showed that Ti-SLA was the highest, followed by zirconia and Ti-turned surfaces. The mean Sdr value for one surface was significantly different from that for another (*p* < 0.05). Error bars represent the standard deviation. (**) represents the significance, ** *p* < 0.05.

Figure 2 shows the data for the roughness parameters (Sa, Sdr) of the groups. The means for Sa were 0.65 μm (0.05 μm) for Ti-SLA, 0.27 μm (0.05 μm) for Ti-turned, and 0.54 μm (0.03 μm) for the zirconia implants. The surfaces of the Ti-SLA and zirconia implants were significantly rougher than that of Ti-turned implants based on the Sa value (*p* < 0.01). There was no significant difference between the Ti-SLA and zirconia implants for Sa (*p* = 0.084). The means for Sdr were 165.22% (16.39%) for Ti-SLA, 23.01% (6.49%) for Ti-turned, and 106.93% (6.32%) for zirconia. The mean Sdr values showed that Ti-SLA was the highest, which was followed by zirconia and Ti-turned surfaces. The mean Sdr value for one surface was significantly different from that for another (*p* < 0.05).

The results of ESCA analysis are shown in Table 1. Titanium (Ti), nitrogen (N), and oxygen (O) were confirmed in Ti implants. Zirconium (Zr), hafnium (Hf), aluminum (Al), yttrium (Y), and oxygen (O) were detected in zirconia implants.

### 3.2. Histologic and Histomorphometric Analysis

**Figure 3:**
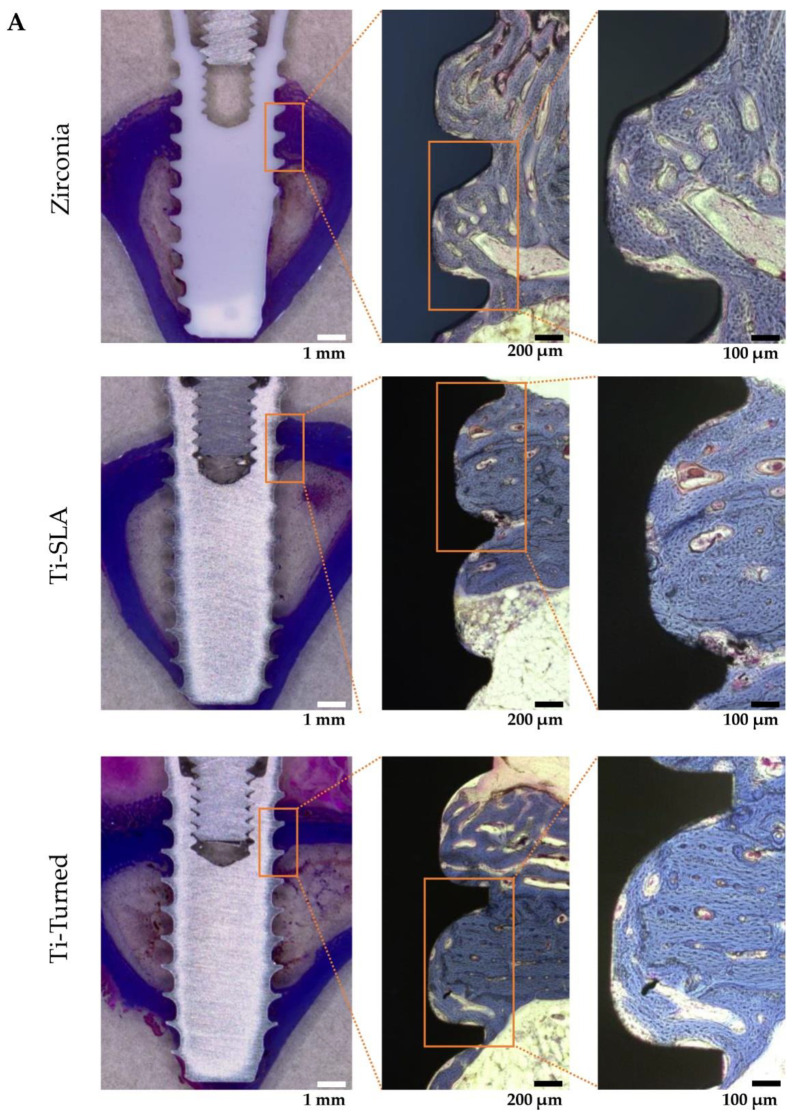

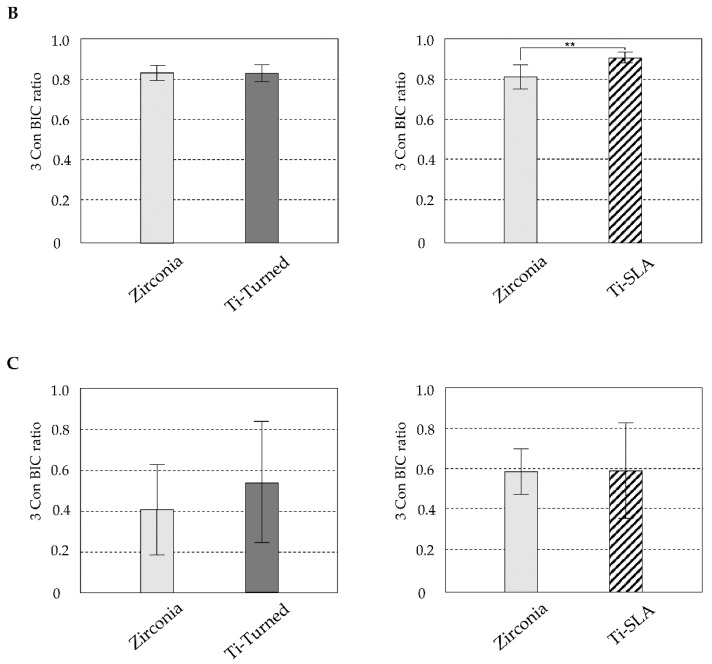
(**A**) Representative light microscopy photomicrographs of implants placed into rabbit tibia after 28 days from the installation. Zirconia (top), Ti-SLA (middle), and Ti-Turned implants (bottom) (magnification ×12.5, ×50, and ×100 from the left, Masson Trichrome staining, scale bar on lower right); (**B**) Bone-to-implant contact ratios were calculated at the best three consecutive threads on both sides of the sectioned implant; (**C**) Bone area ratios were measured and defined as the ratio of the osseous area to the total area between interested implant threads. Data are expressed as the mean ± SD (*n* = 3). Error bars display the standard deviation. (**) represents the significance, ** *p* < 0.05.

The mature bone and newly formed bone might be distinguished by the Masson’s trichrome staining method [25]. In the cortical area, where the neck and apex of the implants were placed, the existing mature bones were stained blue (Figure 3A). In the histological analysis of zirconia, Ti-turned, and Ti-SLA implants, bone formation was found in peri-implant areas. Highly dense bone was especially visible in the Ti-SLA implant, along the bone–implant interface. New immature bone was detected and stained red at the implant threads and around the mature bone (Figure 3A). The mean values and standard deviation (SD) for BIC (%) were 83.20 ± 4.06 for Ti-turned and 83.39 ± 3.52 for zirconia in the two experimental animals. Additionally, in the remaining animals, 90.52 ± 2.64 was found for Ti-SLA, and 81.19 ± 5.79 was found for zirconia for the mean values and SD of BIC (%) (Figure 3B). The mean values and SD for BA (%) were 53.22 ± 29.66 for Ti-turned and 40.58 ± 21.98 for zirconia, while those were 59.24 ± 23.20 for Ti-SLA and 58.85 ± 11.02 for zirconia (Figure 3C). The zirconia and Ti-SLA implants showed a significant difference in BIC (*p* = 0.012), but not in BA (*p* > 0.05). There were no significant differences between Ti-turned and zirconia implants in either BIC or BA (*p* > 0.05).

## 4. Discussion

The topography and chemistry of implant in the bone are closely related to the reaction of the tissues surrounding the implants [26,27]. The present study took place over 4 weeks, with respect to BIC% and BA%, revealing significantly high values of Ti-SLA implants in comparison to zirconia and Ti-turned implants in tibia. Ti-turned and zirconia implants showed similar values, which were not significantly different from each other, in BIC%. The results indicated that the zirconia implant seemed to have a similar biocompatibility to commercially pure titanium implant.

Bone-to-implant contact (BIC%) and peri-implant bone area (BA%) are two important variables in quantitative analysis when evaluating the degree of osseointegration and discriminating between implant designs, surface composition, or surface modification [28,29]. In the rabbit model, Scarano et al. [30] reported that the untreated zirconia implant had a mean BIC of 68% after 4 weeks of placement and demonstrated that untreated zirconia implants showed considerable biocompatibility. Gehrke et al. reported very similar BIC values between the titanium (machined and treated surface) and zirconia implants, without any statistically significant differences between the three groups, similar to other research [31].

The implant surface modification enhances bone integration, which, in animal studies, is observed as higher BIC [32,33]. Sennerby et al. demonstrated that the surface-modified zirconia implant had a stronger bone tissue response compared with the machined zirconia implant [34]. These are consistent with the findings of other studies, where Mihatovic et al. presented roughened surface zirconia implants in dogs, which showed a higher BIC than the machined zirconia implants after ten weeks of healing [11]. However, in these studies, there were no significant differences between surface-modified zirconia and untreated zirconia [11,34]. Similarly, as the bone and tissue responses of the Ti-SLA implant were higher than those of the untreated titanium implant [35,36], it might be estimated that proper surface treatment with zirconia implants will enhance the osseointegration process. Through the surface physical test of this study, it was confirmed that the zirconia, Ti-turned, and Ti-SLA implants had different surface topography and roughness parameters. However, the results were not consistent with the in vivo histomorphometric values in this study. Since the physicochemical properties of materials could cause variable cell reactions [37], it was difficult to determine a causal relationship clearly.

The zirconia implants used in previous studies were usually manufactured by the conventional subtractive method, milling out of zirconia blanks. However, this subtractive manufacturing method leads to several problems, including the expensive molds and severe wear of cutting tools, limited reproduction of surface geometry, as caused by the predetermined size and shape of the milling instruments, and the axis of the computer numerical controlled (CNC) machine with a confined range of operation. Flaws such as cracking might also be created during the manufacturing process [17,38,39,40,41]. However, 3D printing enables the preparation of highly complex and elaborate structures and the production of many objects in a single run [42]. By applying 3D-printing techniques, it is possible to remove the residual stress that is commonly seen in the traditional digital milling process and to increase the precision to separate the implant and abutment, as in the titanium implant system. Furthermore, the advantage of producing a product in a single run might be that it provides a flexible approach to the zirconia surface-treatment method. To the best of our knowledge, this study first evaluated the hard tissue response to the 3D-printed zirconia implant surface, which requires a certain modification to enhance its biocompatibility, in vivo. 

The time of 4 weeks determined in this study was based on the previous literature [43,44], which demonstrated that the bone remodeling process is terminated for the rabbit animal model. There is a need to further evaluate the effect of early bone response in surface-treated 3D-printed zirconia implants, which requires experimental designs with varying sacrifice times and larger sample sizes. The 3D-printed zirconia implants in this study were identical to the titanium implants in their macro- and micro-structure. Although the design of the zirconia implant is identical to that of the titanium implant, a mismatch between the drilled hole and the zirconia implant might have arisen due to production-related inaccuracies. Thus, these minor mismatched aspects might lead to a compromised bone response when using zirconia implants. Furthermore, it is assumed that aforementioned diverse pores, imperfections, flaws, or microcracks entrapped during the lithography-based ceramic manufacturing process lower the mechanical strength of the 3D-printed zirconia implant [45], and when bending force or cyclic loading is additionally applied, this can lead to early implant failure [46]. Therefore, further studies are needed to investigate the effect of aging, such as thermocycling and cyclic loading, on the strength of the 3D-printed zirconia implants.

## 5. Conclusions

Additively manufactured zirconia ceramic implants are biocompatible at the level of commercially pure titanium implants, which have been reported to be successful for long-term clinical service. However, this study showed that the 3D-printed surface of these zirconia implants was inferior to that of the SLA titanium implants that are globally used in clinics for faster and stronger osseointegration. Considering the results of this in vivo study, an adequate modification method needs to be developed for the clinical application of these 3D-printed zirconia implants.

## Figures and Tables

**Table 1:** Elemental composition analysis of printed implants and Ti implants were performed using electron spectroscopy for chemical analysis (ESCA).

Atomic Conc (%).	Hf	Al	Y	Zr	O	Ti	N
Zirconia ^1^	0.35 ± 0.17	1.63 ± 0.17	2.09 ± 0.28	12.33 ± 1.67	83.60 ± 1.98		
Ti-SLA ^2^					72.61 ± 0.25	26.57 ± 0.26	0.82 ± 0.02
Ti-Turned ^3^					73.97 ± 0.05	23.44 ± 1.29	2.60 ± 1.24

^1^ Zirconia = zirconia implant without surface modification; ^2^ Ti-SLA = sandblasted, large-grit, acid-etched surface; ^3^ Ti-turned = commercially pure titanium without surface modification.

## Data Availability

Not applicable.
